# Neurobiology of Alzheimer's disease

**DOI:** 10.4103/0019-5545.44908

**Published:** 2009

**Authors:** E. Mohandas, V. Rajmohan, B. Raghunath

**Affiliations:** Department of Psychiatry, Elite Mission Hospital, Thrissur, Kerala, India; 1 Department of Neurology, Elite Mission Hospital, Thrissur, Kerala, India

**Keywords:** Alzheimer's disease, amyloid cascade, tau hyperphosphorylation, vascular hypothesis, cholinergic deficit

## Abstract

Alzheimer's disease (AD) is a devastating neurodegenerative disease, the most common among the dementing illnesses. The neuropathological hallmarks of AD include extracellular β-amyloid (amyloid precursor protein (APP) deposits, intracellular neurofibrillary tangles (NFT)), dystrophic neuritis and amyloid angiopathy. The mismetabolism of APP and the defective clearance of β amyloid generate a cascade of events including hyperphosphorylated tau (τ) mediated breakdown of microtubular assembly and resultant synaptic failure which results in AD. The exact aetiopathogenesis of AD is still obscure. The preeminent hypotheses of AD include amyloid cascade hypothesis and tau hyperphosphorylation. The amyloid hypothesis states that extracellular amyloid plaques formed by aggregates of Aβ peptide generated by the proteolytic cleavages of APP are central to AD pathology. Intracellular assembly states of the oligomeric and protofibrillar species may facilitate tau hyperphosphorylation, disruption of proteasome and mitochondria function, dysregulation of calcium homeostasis, synaptic failure, and cognitive dysfunction. The tau hypothesis states that excessive or abnormal phosphorylation of tau results in the transformation of normal adult tau into PHF-tau (paired helical filament) and NFTs. Vascular hypothesis is also proposed for AD and it concludes that advancing age and the presence of vascular risk factors create a Critically Attained Threshold of Cerebral Hypoperfusion (CATCH) which leads to cellular and subcellular pathology involving protein synthesis, development of plaques, inflammatory response, and synaptic damage leading to the manifestations of AD. Multiple other aetiological and pathogenetic hypotheses have been put forward including genetics, oxidative stress, dysfunctional calcium homeostasis, hormonal, inflammatory-immunologic, and cell cycle dysregulation with the resultant neurotransmitter dysfunctions and cognitive decline. The available therapeutic agents target only the neurotransmitter dysfunction in AD and agents specifically targeting the pathogenetic mechanisms like amyloid deposition and tau hyperphosphorylation might provide a definite therapeutic edge.

## INTRODUCTION

Alzheimer's disease (AD) is devastating neurodegenerative disease, the most common among the dementing illnesses. The neuropathological hallmarks of AD include extracellular β-amyloid (amyloid precursor protein (APP) deposits, intracellular neurofibrillary tangles (NFT)), dystrophic neuritis, and amyloid angiopathy. The mismetabolism of APP of unknown etiology (genetic factors contribute only a minority of familial AD) and the defective clearance of β amyloid generate a cascade of events including hyperphosphorylated tau (τ) mediated breakdown of microtubular assembly. This results in synaptic failure. Initial glutamatergic deficit at the transentorhinal region paves the way for degenerative changes in the hippocampus and amygdala. In latter stages, the neuronal destruction is manifest in parietal and frontal cortices. Multiple aetiological and pathogenetic hypotheses have been put forward including genetics, oxidative stress, dysfunctional calcium homeostasis, hormonal, inflammatory-immunologic, vascular, and cell cycle dysregulation with the resultant neurotransmitter dysfunctions and cognitive decline. However, amyloid cascade hypothesis along with the tau hyperphosphorylation is still the most proposed pathogenetic mechanism.[1]

## AMYLOID CASCADE HYPOTHESIS

The amyloid hypothesis states that amyloid plaques formed by aggregates of Aβ peptide generated by the proteolytic cleavages of APP are central to AD pathology.[1] APP belongs to a large family of type I membrane proteins with a large extracellular domain and a short cytoplasmic region derived by differential splicing of a single gene transript located on the long arm of chromosome 21. The predominant isoforms, APP770, APP751, and APP695 are expressed with some tissue specificity. The two longer isoforms of APP, APP751 and APP770, contain a 56 amino acid long ectodomain. APP is cleaved throughout the Golgi complex by O-glycosylation. The major processing pathway of APP is nonamyloidogenic, the cleavage necessitated by ά-secretase occurring between Lys16 and Leu17 within the Aβ domain preventing the formation of Aβpeptides. During this cleavage a soluble ectodomain of APP (sAPPά) is released and a 10-kDa C-terminal fragment (p3CT) remains within the membrane.[2,3]

The soluble peptide derived from APP, sAPPά may have neuroprotective roles. At least 30% of APP is processed by this pathway.[4,5]

**Figure 1:**
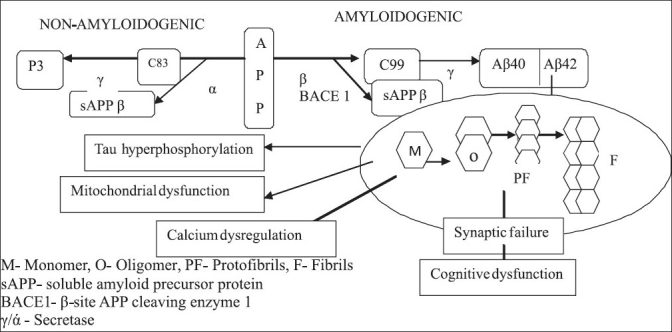
Amyloid cascade hypothesis

Aβ generation from APP occurs via a two-step proteolytic process involving β- and γ-secretases. The β-site APP cleaving enzyme (BACE1), first cleaves APP to generate a membrane bound soluble C-terminal fragment. A subsequent cleavage of the C-terminal fragment by the γ-secretase activity further generates Aβ_40_ and Aβ_42_.[2] Both types of peptide could be found in amyloid plaques, but Aβ_42_ is apparently more directly neurotoxic and has a greater propensity to aggregate.[3] This pathway by which APP is cleaved is called amyloidogenic pathway. Under normal conditions, about 90% of secreted Aβ peptides are Aβ_40_, which is a soluble form of the peptide that only slowly converts to an insoluble β-sheet configuration and thus can be eliminated from the brain. In contrast, about 10% of secreted Aβ peptides are Aβ_42_, species that are highly fibrillogenic and deposited early in individuals with AD and Down's syndrome. Intracellular assembly states of Aβ are monomers, oligomers, protofibrils, and fibrils. The monomeric species are not pathological, however the nucleation dependent fibril formation related to protein misfolding makes the Aβ toxic. The oligomeric and protofibrillar species may facilitate tau hyperphosphorylation, disruption of proteasome and mitochondria function, dysregulation of calcium homeostasis, synaptic failure and cognitive dysfunction[4,5] [Figure 1].

## TAU HYPOTHESIS

The tau hypothesis states that excessive or abnormal phosphorylation of tau results in the transformation of normal adult tau into PHF-tau (paired helical filament) and NFTs. Tau protein is a highly soluble microtubule-associated protein (MAP). Through its isoforms and phosphorylation tau protein interacts with tubulin to stabilize microtubule assembly. Tau proteins constitute a family of six isoforms with the range from 352-441 amino acids. The longest isoform in the CNS has four repeats (R1, R2, R3, and R4) and two inserts (441 amino acids total), whereas the shortest isoform has three repeats (R1, R3, and R4) and no insert (352 amino acids total). All of the six tau isoforms are present in an often hyperphosphorylated state in paired helical filaments from AD.

**Figure 2:**
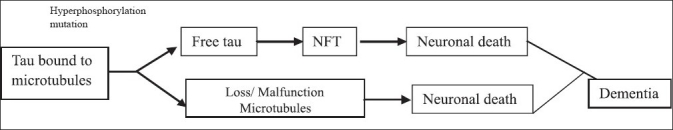
Tau hypothesis

Mutations that alter function and isoform expression of tau lead to hyperphosphorylation. The process of tau aggregation in the absence of mutations is not known but might result from increased phosphorylation, protease action or exposure to polyanions, such as glycosaminoglycans.[6] Hyperphosphorylated tau disassembles microtubules and sequesters normal tau, MAP 1(microtubule associated protein1), MAP 2, and ubiquitin into tangles of PHFs. This insoluble structure damages cytoplasmic functions and interferes with axonal transport, which can lead to cell death.[7] [Figure 2]

## INFLAMMATORY HYPOTHESIS

Microglia, astrocytes and possibly to a lesser extent the neurons are involved in the inflammatory process in AD. Aβ can activate microglia which leads to an increase in cell surface expression of major histocompatibility complex II (MHC II) along with increased secretion of the pro-inflammatory cytokines interleukin-1β (IL-1β), interleukin-6 (IL-6), and tumor necrosis factor α (TNF α) as well as the chemokines- interleukin-8 (IL-8), macrophage inflammatory protein-1 α (MIP-1 α), and monocyte chemo-attractant protein-1.[8] Aβ also induces a phagocytic response in microglia and expression of nitric oxide synthase (NOS) resulting in neuronal damage.[9] Microglia may also play a role in the degradation of Aβ by the release of insulin degrading enzyme (IDE).

Astrocytes also cluster at sites of Aβ deposits and secrete interleukins, prostaglandins, leukotrienes, thromboxanes, coagulation factors, and protease inhibitors. Neurons themselves are able to express significantly higher levels of classical pathway complement and pro-inflammatory products that trigger inflammatory processes. Further, the complement system, cytokines, chemokines, and acute phase proteins (especially pentraxins) contribute to the inflammatory response in AD. The neuroinflammation as a primary cause or secondary effect in Alzheimerogenesis is a chicken and egg question.[10]

## OXIDATIVE STRESS HYPOTHESIS

Reactive oxygen species (ROS or free radicals) the major portion of which (95-98%) comes from the byproducts of the electron transport chain (ETC) of the mitochondria, may mediate oxidative cell injury and cell death.[11] Mitochondrial oxidative phosphorylation is the major source of free radicals like the hydrogen peroxide radicals (H_2_O_2_), hydroxyl radicals (OH·) and the superoxide radical (O_2_^-.^). The oxidative damage that ensues is seen in lipids, proteins, nucleic acids, and sugars all of which are organic compounds essential for the structural and functional integrity of neurons.[12]

In AD, there is inhibition of ETC resulting in the accumulation of electrons in the complex I and coenzyme Q (CoQ). The electrons therein accumulated can be donated directly to molecular oxygen to form the superoxide radical (O_2_^-.^) The superoxide radical (O_2_^-.^) can react with nitric oxide to form peroxynitrate radical (OONO^-^). The H_2_O_2_ in the presence of transition metals is converted to the toxic hydroxyl (OH·) radical. Therefore, AD is associated with dysfunction of the ETC and free radical production. AD is also characterized by a deficiency of antioxidant capacity. The activities of the enzymes Cu/Zn SOD (superoxide dismutase) are reduced and there is a deficiency of glutathione (GSH). The free radicals thus are able to produce cellular damage unchecked by antioxidants.[13]

ROS mediated DNA oxidation causes strand breaks, DNA-protein cross linking and base modification. Glyco-oxidation by ROS results in the formation of advanced glycation end products (AGEs). AGE modified protein can produce more ROS. There is direct biochemical link between AGEs and lipid peroxidation leading to further advancement of AD pathology.[13]

The mitochondrial hypothesis, the hydrogen peroxide hypothesis and the amyloid beta synergistic endothelial and neuronal toxicity (ABSENT) hypothesis revolve around ROS mediated neuronal dysfunction either directly or indirectly.

## VASCULAR HYPOTHESIS

**Figure 3:**
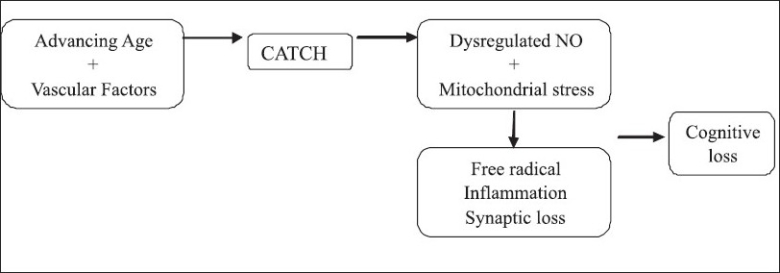
Vascular hypothesis

The vascular hypothesis proposes that AD develops when two biological events converge namely advancing age and the presence of vascular risk factors for AD. Epidemiological data exist implicating vascular risk factors in AD. Shared risk factors in both AD and vascular dementia and the response to pharmacotherapy targeting vascular factors benefiting AD are additional findings. Cerebral microvascular pathology and cerebral hypoperfusion may trigger the cognitive and degenerative changes in AD. Advancing age and the presence of vascular risk factors create a critically attained threshold of cerebral hypoperfusion (CATCH). CATCH is an unremitting and progressive pathology affecting cerebral capillaries. When CATCH is reached, there is dysregulation of endothelial nitric oxide (NO) production leading to capillary degeneration. This along with the lowered ATP or energy supply leads to mitochondrial oxidative stress. The resulting crisis leads to cellular and subcellular pathology involving protein synthesis, development of plaques, inflammatory response, and synaptic damage leading to the manifestations of AD[14] [Figure 3].

## CHOLESTEROL HYPOTHESIS

Hypercholesterolaemia is a modifiable risk factor in AD. ApoE is the major apolipoprotein in the brain, modifies the age of onset for AD. Apolipoprotein E4 (ApoE4) is essential for the normal catabolism of triglyceride-rich lipoprotein constituents. It is a target gene of liver X receptor, a nuclear receptor member that play role in metabolism of cholesterol, fatty acid, and glucose homeostasis. ApoE4 homozygotes have a mean age of onset of <70 years compared to >80 years for ApoE3 homozygotes. In contrast, inheritance of one ApoE2 allele delays the age of onset of AD to >90 years.[15]

The precise mechanisms by which ApoE participates in AD pathogenesis remain largely undefined. Several hypotheses are proposed to explain the same they include (a) the proposed role of ApoE to mediate neuroinflammation, (b) its participation in the regulation of the cholinergic neurotransmitter system, (c) its role in neuronal signaling, and (d) ApoEs maintenance of the integrity of the blood–brain barrier. However, the most prominent hypotheses of ApoE function is its key role as a mediator of Aβ metabolism. ApoE binds Aβ, affects the deposition and clearance of Aβ, and is required for amyloid deposition. Furthermore, ApoE affects amyloid deposition in an allele-specific manner. However, the exact pathophysiologic process is yet to be elucidated.[16]

The role of cholesterol in the pathology of AD is also shown by the ability of statins to reduce the prevalence of AD by up to 70%. Similarly, inhibition of cholesterol biosynthesis by statins and another cholesterol synthesis inhibitor was found to reduce amyloid burden in guinea pigs and murine models of AD. However, several prospective studies have demonstrated that statins reduce the turnover of brain cholesterol at standard therapeutic doses, although the steady-state levels of Aβ in the cerebrospinal fluid (CSF) remain unaltered. The role of statins in AD is still a subject for further investigation.[17]

The role of cholesterol is further brought to the fore by the fact that intracellular cholesterol may regulate APP processing by directly modulating secretase activity or by affecting the intracellular trafficking of secretases and/or APP. Cholesterol loading increases γ-secretase activity and amyloidogenic pathway while low intracellular cholesterol favors non-amyloidogenic pathway. There are also genetic factors linking cholesterol metabolism and AD, though ApoE is the only gene with replicable evidence, several candidate genes involved in lipid metabolism like A2M (alpha-2-Macroglobulin), LRP (lipoprotein receptor related protein), IDE (insulin degrading enzyme), ABCA1 (ATP-binding cassette transporter), ACAT (Acyl-CoA cholesteryl acyl transferase), CYP 46 (Cytochrome P450, family 46, convertscholesterol to 24S-hydroxycholesterol) are being investigated for putative roles with mixed results.[17]

Biochemical and pharmacological evidence strongly support a role for cholesterol and lipid metabolism in Aβ generation, deposition, and clearance. With the exception of ApoE, further candidate genes need to be identified.[17]

## METALLOBIOLOGY

High levels of copper (Cu), iron (Fe), and zinc (Zn) are seen in the amyloid plaques and both Aβ and APP have metal ion binding sites. Micromolecular concentrations of Zn^2+^ can result in the precipitation of β amyloid at physiologic pH. Age dependent hyperactivity of the ZnT3 transporter in females accounts for the increased incidence of AD. AβCu complexes and plaques that are bound to Zn may reduce H_2_O_2_ production and resultant toxicity than soluble amyloids that are not bound to Zn.[18]

Micromolecular concentrations of Cu and Fe on the other hand precipitate β amyloid at slightly acidic pH. Cu and Fe are also co-factors to β amyloid in the generation of oxidative stress. Aβ via its binding to Cu^2+^ (AβCu^2+^) and Fe^3+^ (Aβ Fe^3+^) converts them to Cu^+^ and Fe^2+^, respectively and this is followed by the generation of H_2_O_2_ by double electron transfer to oxygen. This generation of H_2_O_2_ creates the ideal milieu for the generation of highly reactive hydroxyl radicals (OH·) (Fenton reaction). AβCu^2+^ is oxidized by H_2_O_2_ to form cross-linked and soluble forms of Aβ. Oxidized Aβ oligomers are resistant to proteolysis and they are likely to become subunits for Zn^2+^ induced assembly to amyloid mass.[18]

The management of metal ions in the brain is by protein and transport systems like the metallothionien system and by genetic factor proteins like apolipoprotein E_2_ and α2- macroglobulin all of which are reported to be abnormal in people with AD. Further abnormalities are described in the blood brain barrier (BBB), a key structure that resists the transduction to the brain of plasma metal ion level changes. All of these could result in an increased pooling of metal ions.[18]

The Aβ and APP oppose the elevation of metal ions. The Aβ at high peptide to metal stoichiometry is protective by removing metal ions, while at high metal ion to peptide stoichiometry it becomes aggregated and oxidized. Thus, metals like Zn, Cu, and Fe interact in a complex manner with β amyloid. However, the efficacy of chelating agents like clioquinol in AD has been questioned.[18]

## INSULIN SIGNALING

Abnormal function of the insulin/insulin-like growth factor I (IGF-I) axis may be another putative mechanism in AD. Both insulin and IGF-1stimulate Aβ release from neurons, and IGF-I exerts a stimulatory effect on brain amyloid clearance. In addition, insulin and IGF-I levels are altered in Alzheimer's patients and, probably cell sensitivity towards insulin and possibly IGF is decreased in these patients. Insulin exerts a double-sided effect on brain β amyloid. It stimulates neuronal release of β amyloid and at the same time contributes to extraneuronal accumulation of β amyloid by competing for insulin degrading enzyme (IDE). The net action of insulin is therefore to increase brain β amyloid. IGF-I decreases brain levels of β amyloid and increases plasma levels of β amyloid complexed to transport proteins. Thereby IGF-I stimulates clearance of brain β amyloid. A stepwise loss of sensitivity to IGF-I and then to insulin leads to brain accumulation of β amyloid. Initially reduced sensitivity to blood-borne IGF-I at BBB causes reduced clearance of β amyloid, causing brain accumulation of β amyloid. Increased levels of Aβ antagonize insulin and IGF-I binding to their corresponding receptors, which induces a resistant state to insulin/IGF-I in neurons. In response to this resistant state, a homeostatic compensatory mechanism develops whereby levels of insulin/IGF-I increase in an attempt to rescue loss of function on target cells. High levels of insulin diminish availability of IDE to degrade β amyloid and as a result more β amyloid accumulates and establishes a self-perpetuating vicious circle.[19]

## CELL CYCLE HYPOTHESIS

Cell cycle hypothesis proposes that either mitogenic signaling, or cell cycle control, or both, are deranged in AD. Ectopic expression of cell cycle molecules as cdc2 (cell division cycle 2), cdk4 (cyclin dependent kinase 4), and others have been reported in vulnerable neurons in AD. Vulnerable neurons in AD brain reenter the cell cycle. APP has a role in the activation of neuronal cell cycle proteins and a failure of regulation of this pathway occurs in neurons in AD brain. The modest overexpression of familial AD mutants of APP results in (1) an increase in expression of APP-BP1 (APP binding protein I) in lipid rafts, (2) entry of the neurons into the S phase of the cell cycle, and (3) neuronal apoptosis. The interaction of APP with APP-BP1 activates a pathway leading to the conjugation of NEDD8, an ubiquitin-like protein. Proteins known to be neddylated via this pathway are a family of proteins called cullins. Cullins are scaffold proteins and neddylation of cullin enhances its ability to promote ubiquitination. The neddylation pathway promotes FAD (familial AD) APP-mediated cell cycle entry (drives the cell cycle through the S–M checkpoint) and apoptosis.[20] Another cell cycle mediator important in AD is GSK3 (glycogen synthase kinase). GSK3 plays a role in both sporadic and familial forms of AD and over-activity of GSK3 accounts for memory impairment, tau hyper-phosphorylation, increased β-amyloid production, and inflammatory responses. Activation of PPARγ (peroxisome proliferator activated receptor γ) is also implicated in cell cycle as it inhibits the generation of proinflammatory and neurotoxic products in microglia and monocytes exposed to b-amyloid. PPARγ agonists decrease the secretion of β-amyloid and attenuate Aβ-mediated impairment of long-term potentiation.[20]

Additionally, member of the cyclin-dependent kinase (cdk) family have been implicated in tau hyperphosphorylation and the consequent development of neurofibrillary tangles in AD. Concomitant activation of cdc2, cdk4, and cdk5 in neurodegenerating neurons has been described in AD and concurrent p25 accumulation, cdk5 activation, and tau hyperphosphorylation were also observed in the postmortem brains of AD patients. The regulation of cell cycle by Pin 1 (peptidyl-prolyl isomerases which catalyzes the isomerization of the peptide bond between pSer/Thr-Pro in proteins), is implicated in AD. In dividing cells, Pin1 regulates the progression of the cell cycle by interacting with a large number of mitosis-specific phosphoproteins. Pin1 might facilitate the transition from G0 to G1 (cell cycle checkpoints) in neurons, leading eventually to neuronal dedifferentiation and apoptosis.[21]

## NEUROTRANSMITTERS IN ALZHEIMER's DISEASE

### Acetylcholine

Basal forebrain cholinergic cell loss is a consistent feature of AD. Impaired cortical cholinergic neurotransmission contributes to Aβ pathology and increases phosphorylation of tau protein. Selective activation of M1-M3-but not M2-M4-mAChR (muscarinic acetyl choline receptor) increases sAPPα secretion and decreases total Aβ formation. The mAChRs mediate their effects on APP processing through activation of the phosphatidyl inositol signaling pathway and probably via the tyrosine kinase MAP (mitogen activated protein) kinase pathway. In contrast, BACE1 expression was downregulated by activation of M2-mAChR and protein kinase A-mediated pathways. Nicotine through action on nicotinic nAChR (nicotinic acetyl choline receptor) has also been observed to modulate APP processing by favoring the non-amyloidogenic pathway. Nicotine also causes inhibition of Aβ fibril formation and disruption of preformed Aβ fibrils. Aβ decreases the intracellular acetylcholine concentration and impairs M1 receptors. By direct binding with high affinity to nAChR, in particular to the α7 subtype, Aβ may disrupt the receptor function.[21,22] Activation of nAChR results in a significant increase in tau phosphorylation, whereas mAChR activation may prevent tau phosphorylation.[23]

### Glutamate

Malfunctions in components of the glutamate–glutamine cycle could result in a self-perpetuating neuronal death cascade and glutamatergic excitotoxicity.[24] Chronic neuronal insults may lead to the activation of extra-synaptic NMDA (N-methyl D aspartate) receptors. This interacts with ‘fyn’ [src (proto oncogenic) family tyrosine kinase] through two scaffolding proteins, DLG4 (Discs large homolog 4, involved in anchoring synaptic proteins) and GNB2L1 (Guanine nucleotide binding protein). Mechanisms of DLG4 association and GNB2L1 dissociation from Fyn contribute to chronic NMDAR hyperactivity in AD. Fyn further activates extra-synaptic NMDA (NR2B subunit) causing continued Ca^+2^ influx into the cytoplasm. High intracellular Ca^+2^ lead to mitochondrial dysfunction. Chronic over-activation of extra-synaptic NMDAR sends a CREB (cyclic AMP response element binding protein) shut-off signal whereby levels of phospho-CREB decline. This leads to decreased production of pro-survival signals like BDNF (brain derived neurotrophic factor). All these events lead to cellular dysfunction and neuronal death over a period of time. Aβ via stimulation of nitric oxide enhances glutamate release. Aβ also inhibits glial uptake of glutamate and thus contributes to glutamatergic excitotoxicity.[25]

GluR2 subunit of the AMPA is involved in the neuronal death cascade and their overproduction confers a protective advantage. The loss of the GluR2 can influence neuronal susceptibility to Ca^2+^-mediated neurotoxicity leading to neurodegeneration. There is also evidence for impaired glutamate uptake due to deficiency of glial transporters in AD. The interaction between Aβ, glutamate transporters, and oxidative radicals is seen in AD.[24]

#### Serotonin

Serotoninergic involvement in AD is evidenced by the observations that (a) AD is characterized by CSF alteration of 5HT and 5HIAA (b) loss of 5HT synthesizing neurons and 5HT receptors in AD (c) the presence of 5 HT polymorphisms in AD and (d) the improvement especially of agitation and other behavioral symptoms of AD with serotonergic agents.[26]

### Norepinephrine

There is loss of locus coeruleus (LC) neurons in AD. Norepinephrine in several brain areas is reduced and this reduction is mostly limited to patients with an earlier age of and greater severity of intellectual deterioration. There is loss of α-2 receptor function in AD. Findings also show that an intact noradrenergic system is a prerequisite for the integrity of at least some central cholinergic functions. Therefore, multiple lines of evidence implicate norepinephrine in AD.[27]

### Dopamine

There is evidence that micromolar concentrations of dopamine or L-dopa are sufficient to significantly inhibit fibril formation or disaggregate existing fibrils of Aβ. D1 receptor seems to play a more prominent role in mediating plasticity and specific aspects of cognitive function, including spatial learning and memory processes and D1 agonists are being tried for improving cognition in AD.[27]

### Others

There is also emerging evidence that neuropeptides/modulator systems and estrogen are likely to play a role in memory dysfunction or AD. The role of these as possible therapeutic targets is an area of active research.[24]

## CONCLUSION

The exact aetiopathogenesis of Alzheimer's disease (AD) is still obscure. Neurobiological mechanisms probably involved in AD include hypotheses explaining the genesis of excitotoxic fibrillar Aβ and defective clearance of toxic amyloid. The proposed mechanisms include amyloid cascade hypothesis, tau hyperphosphorylation, vascular and oxidative factors, and cell cycle aberrations. The available therapeutic agents target only the neurotransmitter dysfunction in AD. Agents which interfere with cleavage (BACE 1 Inhibitor), reduce aggregation of Aβ, and accelerate the clearance of neurotoxic amyloid may provide better cognitive advantage in AD.
